# Collagen-Coated Hyperelastic Bone Promotes Osteoblast Adhesion and Proliferation

**DOI:** 10.3390/ma16216996

**Published:** 2023-11-01

**Authors:** Andrei Gresita, Iman Raja, Eugen Petcu, Michael Hadjiargyrou

**Affiliations:** 1 Department of Biomedical Sciences, College of Osteopathic Medicine, New York Institute of Technology, Old Westbury, NY 11568, USA; agresita@nyit.edu (A.G.); iraja@nyit.edu (I.R.); epetcu@nyit.edu (E.P.); 2 Department of Biological & Chemical Sciences, New York Institute of Technology, Old Westbury, NY 11568, USA

**Keywords:** hyperelastic bone, 3D-printed scaffold, degradation, biocompatibility, hydroxyapatite, osteogenic differentiation, MG-63 cells, poly-lactic-co-glycolic acid, PLGA

## Abstract

Successfully reconstructing bone and restoring its dynamic function represents a significant challenge for medicine. Critical size defects (CSDs), resulting from trauma, tumor removal, or degenerative conditions, do not naturally heal and often require complex bone grafting. However, these grafts carry risks, such as tissue rejection, infections, and surgical site damage, necessitating the development of alternative treatments. Three-dimensional and four-dimensional printed synthetic biomaterials represent a viable alternative, as they carry low production costs and are highly reproducible. Hyperelastic bone (HB), a biocompatible synthetic polymer consisting of 90% hydroxyapatite and 10% poly(lactic-co-glycolic acid, PLGA), was examined for its potential to support cell adhesion, migration, and proliferation. Specifically, we seeded collagen-coated HB with MG-63 human osteosarcoma cells. Our analysis revealed robust cell adhesion and proliferation over 7 days in vitro, with cells forming uniform monolayers on the external surface of the scaffold. However, no cells were present on the core of the fibers. The cells expressed bone differentiation markers on days 3 and 5. By day 7, the scaffold began to degrade, developing microscopic fissures and fragmentation. In summary, collagen-coated HB scaffolds support cell adhesion and proliferation but exhibit reduced structural support after 7 days in culture. Nevertheless, the intricate 3D architecture holds promise for cellular migration, vascularization, and early osteogenesis.

## 1. Introduction

Effective bone regeneration is imperative in contemporary clinical practice due to the rising prevalence of skeletal fractures generated by trauma, congenital anomalies, revision joint arthroplasty, or tumor resection [1]. Anatomic regions, such as the craniofacial area or extremities, are mostly affected [2]. Considered to be a “Gold Standard” in the treatment of such conditions, autologous bone graft prevails as it provides an osteoinductive scaffold coupled to signaling molecules and osteogenic cells directly to the damaged site [3,4]. Recent data show that more than two million bone grafts are implanted annually worldwide, with one in every four procedures being performed in the US [4,5,6,7]. However, while recent medical advances have brought significant improvements in the orthopedic field, successfully grafting bone tissue still ranks amongst the most challenging clinical procedures [8]. Substantial disadvantages, namely the limited quantity of grafts, postoperative pain, fractures at the harvest site, nerve damage, donor site infection, and socioeconomic costs are associated with this procedure [4,9,10,11]. Further, adequate healing and complete regain of function are dependent on the patient’s age, comorbidities, physical condition, and disease severity [12]. The emergence of post-graft complications, such as medication-related osteonecrosis of the jaw (MRONJ), presents a significant problem, particularly in patients undergoing medical treatments involving anti-resorptive, anti-angiogenic, or immunomodulatory agents [13]. For example, a study that evaluated dental implant therapy in patients with a history of oral bisphosphonate use noted the increased risk of bisphosphonate-related osteonecrosis of the jaw (BRONJ). The study concluded that, despite successful rehabilitation, concerns about BRONJ persist, prompting the recommendation for a case-specific approach to treatment decisions [14]. While traditional dental implants are associated with BRONJ risk, the emergence of 3D and 4D synthetic bone implants offers promise in mitigating complications. These advanced implants are designed to facilitate better integration with the surrounding bone tissue and support more natural bone healing, potentially reducing associated risks. Synthetic biocompatible implants represent a promising alternative for bone repair, as they can be mass-produced at relatively low costs and are easily customizable to fit complex anatomic skeletal defects [15]. Additionally, the precise control of construction and biological and biochemical properties makes these biomimetic scaffolds extremely desirable [15]. Recent studies have proved that biocompatible, nontoxic scaffolds made of chitosan, alginate, collagen, or hyaluronic acid are now able to sustain and promote tissue development while maintaining biomechanical integrity and, ultimately, biodegradability [16]. One such material is represented by hyperelastic bone (HB), which can be printed at high manufacturing rates of up to 27 cm^3^/h from room-temperature extruded liquid inks [17].

A recent study investigated the HB scaffold’s ability to support cell viability, proliferation, and osteogenic differentiation of seeded human mesenchymal stem cells in the absence of osteoinductive factors [17]. Subsequently, HB biocompatibility evaluations were carried out in vivo, encompassing a mouse subcutaneous implant model (7 and 35 days). A rat posterolateral spinal fusion model was also used to assess de novo bone formation (8 weeks), while further evaluation included a rhesus macaque calvaria defect case over a period of 4 weeks. Results unveiled regenerative effects, characterized by the absence of infection, immune rejection, or fibrotic encapsulation. The HB scaffolds demonstrated rapid structural integration with host tissue, ultimately leading to ossification and de novo bone formation [17]. Another study addressed the challenges associated with regenerating large bone fractures by utilizing HB implants enriched with superparamagnetic iron oxide nanoparticles (SPIONs) [18]. The initial phase of the experiment involved in vitro examination, demonstrating the viability of embryonic murine C3H10T12 cells and human-patient-derived osteoblast-like cells seeded on the HB scaffolds over a period of 14 days. Subsequently, in vivo examination, utilizing a rat model of a femoral bone defect proved significant regenerative effects were observed within a two-week timeframe. Importantly, there were no indications of infection, immune rejection, or fibrotic encapsulation [18]. Similar to the previously described study, the HB grafts exhibited rapid integration within the existing tissue and de novo bone formation [17,18]. Another study focused on enhancing regenerative techniques for craniomaxillofacial bone injuries by incorporating a 3D-printed polymer or ceramic-based meshes into mineralized collagen scaffolds [19]. The two mesh types, Fluffy-PLG and HB, were evaluated for their impact on the mechanical and biological properties. Notably, both mesh-reinforced composites demonstrated increased osteogenesis support, upregulating key osteogenic genes like RUNX2, Osterix, and COL1A2. The HB-reinforced composites also exhibited a significant boost in osteoprotegerin secretion, a crucial factor in inhibiting osteoclast-mediated bone resorption [19].

Synthetic bone scaffolds proved substantial efficacy and bone-forming potential, especially when combined with various osteoinductive agents [17,18,19,20]. For example, Wang et al. employed controlled-release recombinant human bone-morphogenetic protein-2 (rhBMP-2) within 3D-printed hydroxyapatite scaffolds to assess its osteogenic potential [17]. Another study that utilized 3D-printed HB demonstrated that the structures exhibited significant osteogenic response in adult human mesenchymal stem cells [21]. Moreover, through sequential seeding of pre-cultured human smooth muscle cells (hSMCs), followed by human umbilical vein endothelial cells (HUVECs), these structures can serve as comprehensive platforms for developing synthetic-based vascularized bone grafts [20]. In vivo experiments also noted successful outcomes, with subcutaneous implantation [22], calvaria defect animal models [23], and even LV-transduced ADSCs implanted in murine models [24]. These findings underscore the considerable translational potential of 3D-bioprinted HB scaffolds alone or enriched with functional nanoparticles [17]. However, it has been reported that the geometry and porosity of 3D-printed HB scaffolds can drastically impact their mechanical characteristics and overall behavior [17,18]. Given these aforementioned studies, we wanted to introduce a variation in the experimental approach using HB, that is, by coating it with collagen to see if it improves its performance as a tissue-engineered scaffold. Herein, we describe our in vitro study using collagen-coated-HB in combination with osteoblastic cells.

In light of these advancements, we hypothesized that the HB scaffold is capable of supporting robust cell adhesion and proliferation. Specifically, our study aimed to assess the biocompatibility, biodegradability, and osteoinductive capacity of a 3D-printed HB scaffold, seeded with MG-63 cells. Considering that hydroxyapatite is the main inorganic component of bone [4], the scaffold was composed of 90% by weight (wt) hydroxyapatite and 10% by weight poly (lactic-co-glycolic acid, PLGA). Initial adhesion experiments involved the use of positive (+collagen) and negative controls (−collagen). However, due to the observation of irregular and non-uniform patterns of cell attachment in the negative control group, we opted to use a type I collagen coating consistently throughout the experimental timeline. Type I collagen promotes cellular adhesion while serving a fundamental role in the native extracellular cellular matrix [25]. Previously, we also functionalized poly(L-lactide) electrospun scaffolds with bioactive collagen molecules and showed that they supported robust osteoblast adhesion and mineralization [26].

## 2. Materials and Methods

### 2.1. Hyperelastic Bone Characteristics

**Figure 1:**
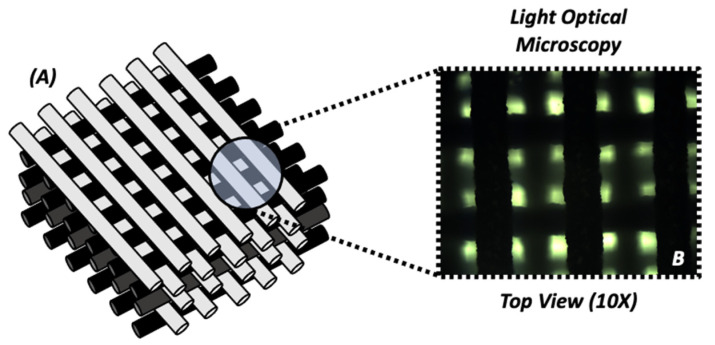
(**A**) Graphical schematic of 3D-printed HE structure. (**B**) Surface morphology observed using light optical microscopy (10×).

The HB scaffold was designed and produced by Dimension Inx LLC, Chicago, IL, USA. The structure is composed of 90% by weight (wt) HA and 10% by weight PLGA, totaling 8 layers at 170 um/layer. The individual fibers are 250 um in diameter with an offset between the fibers of 0–90° and 0–7 mm spacing (Figure 1). HA represents the main ionic component in bone [27] and PLGA is an FDA-approved biodegradable polymer that has been widely used as a scaffold for tissue engineering applications [28,29,30]. Moreover, our laboratory has extensively used PLGA to generate tissue engineering scaffolds designed to deliver DNA [31,32,33,34], growth factors [35], and antibiotics [36], as well as to study their basic properties such as degradation and hydrophilicity [37].

### 2.2. Cell Culture

The MG-63 human osteosarcoma cell line (Sigma Aldrich, St. Louis, MI, USA, 86051601) was cultured in α-MEM (ThermoFisher, Waltham, MA, USA) supplemented with 10% fetal bovine serum (FBS) and 1% penicillin/streptomycin (Sigma-Aldrich) at 37 °C with 5% CO_2_. 

### 2.3. Preparation and Seeding of HB

**Figure 2:**
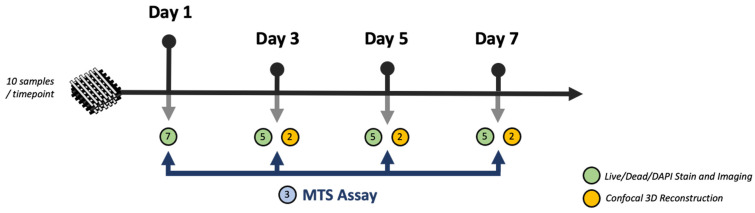
Experimental flowchart illustrating step-by-step sample analysis and workflow.

The 3D-printed HB sheet provided by Dimension Inx LLC was cut into standard-size fragments (7 × 7 mm) and hydrated following the manufacturer’s protocol. To maintain proper sterility, the procedures were performed under a laminar flow hood. Briefly, the individual HB pieces were submerged for 5 min in 70% ethanol, followed by a wash with a phosphate buffer solution (PBS). This ensured both proper hydration as well as the removal of any microscopic fragments that resulted from sectioning. After completion of three hydration and wash cycles, the scaffolds were submerged in rat tail Type 1 collagen (5 mg/mL) for 1 h. Adequate collagen viscosity was maintained by keeping the samples on ice throughout the coating procedure. The excess collagen solution was vacuumed, and the HB samples were transferred into individual wells of a 96-well plate. The scaffolds were then seeded with MG-63 cells, at a concentration of 2.5 × 10^5^ cells/scaffold. Following a 30 min incubation period (37 °C), an additional 150 µL of α-MEM was added to submerge the scaffold in the medium. Figure 2 summarizes the experimental design.

### 2.4. Immunofluorescence and Imaging 

After 1, 3, 5, and 7 days in culture, the cell-seeded scaffolds (*n* = 10/timepoint) were stained with a LIVE/DEAD Viability/Cytotoxicity Kit (ThermoFisher, Waltham, MA, USA). Briefly, the cell-seeded scaffolds were washed with PBS and immersed in LIVE/DEAD solution for 15 min at 37 °C. The staining solution was then removed, and the structures were washed 3X with PBS. Gross cell morphology was also assessed using nuclei stain DAPI (Sigma, St. Louis, MO, USA). Images were taken using a fluorescent microscope (Zeiss Axiovert, Zeiss, Dublin, CA, USA) with Lumenera Infinity 3 camera/software (Teledyne, Waterloo, ON, Canada). Additionally, confocal laser scanning microscopy (CLSM) was used to visualize the proportion and distribution of viable cells on the individual fibers. Cell viability measurements were determined with the help of ImageJ Software 1.49v.

### 2.5. Cell Proliferation Analysis (MTS) 

Cell proliferation, viability, and cytotoxic were assessed using the 3-(4,5-dimethylthiazol-2-yl)-5-(3-carboxymethoxyphenyl)-2-(4-sulfophenyl)-2H-tetrazolium (MTS) assay (ab197010). Prior to the addition of the MTS reagent, the scaffolds were relocated in a sterile 96-well plate, and the reagent was immediately added according to the supplied manufacturer’s protocol. The samples were incubated for 4 h before measuring their absorbance values at 490 nm. The analysis was conducted on days 1, 3, 5, and 7 of the experiment (*n* = 5/timepoint). 

### 2.6. Cryosections

Cell-seeded HB scaffolds (*n* = 3/group) were embedded into Tissue-Tek (Leica, Wetzlar, Germany) and placed in a −80 °C freezer. The blocks were cut, forming 30 µm cross-sections using a cryotome (Leica, Wetzlar, Germany). The cryosections were stained with DAPI for 15 min at 37 °C, followed by a PBS wash and visualized using fluorescent microscopy as described above.

### 2.7. Real-Time Quantitative Polymerase Chain Reaction (qRT-PCR)

Quantitative real-time PCR (qPCR) was used to evaluate the expression levels for the bone differentiation markers, collagen type 1 (Col 1), osteocalcin (OCN), and RUNX-2, with 18S as the housekeeping gene (Table 1). Total RNA was isolated on days 3 and 5 from MG-63 seeded HB scaffolds (*n* = 5/timepoint). The samples were initially homogenized in TriZol reagent (Invitrogen, Waltham, MA, USA), followed by RNA extraction. Sample RNA concentration was measured using the Nanodrop (ND1000). The obtained RNA samples were then used to generate cDNA (High-Capacity cDNA Reverse Transcription Kit from Applied Biosystems, Waltham, MA, USA) per the manufacturer’s instructions. Quantitative real-time PCR (qPCR) was performed on pooled cDNA samples for each time point. Primers were custom designed to amplify sequences within the interest genes Col I, OCN, and RunX-2. All gene expression patterns were normalized to the expression pattern of the housekeeping 18S gene. Reactions utilized the One-Step QuantiTect SYBR Green RT-PCR kit (Qiagen, Hilden, Germany) and were run using a Light Cycler 480 (Roche, Basel, Switzerland). Each experiment was performed 5X to determine the standard deviation. The results were reported as an average fold change relative to each time point ± standard deviation.

### 2.8. Statistical Analyses

All statistical analyses were conducted in GraphPad Prism 9 with one-way analysis of variance (ANOVA). All results are expressed as the mean ± standard deviation. A *p*-value ≤ 0.05 was considered statistically significant. All experiments were conducted in triplicate. The symbols *, **, ***, and **** represent *p* ≤ 0.05, 0.01, and 0.001, 0.0001, respectively. 

## 3. Results

### 3.1. Adhesion 

**Figure 3:**
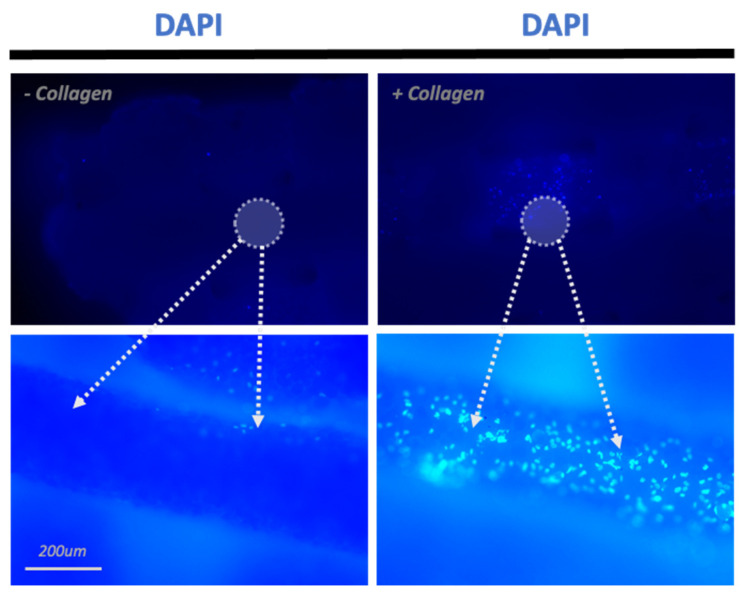
Effect of Collagen. A comparison between non-collagen-coated (−Collagen) and collagen-coated (+Collagen) scaffolds after 1 day in culture using DAPI staining.

In the absence of a collagen coating, cell adhesion was poor on these scaffolds. These uncoated scaffolds demonstrated irregular and non-uniform patterns of cell attachment as opposed to the collagen-based scaffolds that showed robust cell adhesion (Figure 3). These observations underscore the pivotal influence of collagen coating on enhancing cellular adhesion and, subsequently, cellular migration and proliferation, as described below.

**Figure 4:**
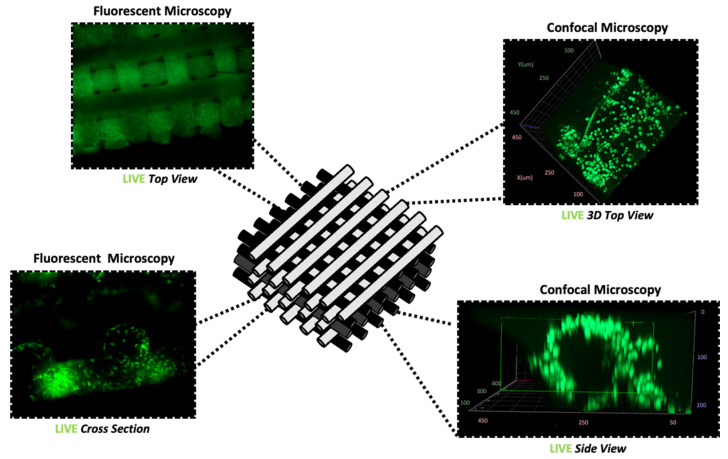
General cellular morphology and distribution evaluated using LIVE stain after 5 days in culture. Fluorescent and confocal microscopy of various angles and sections of the HB.

Throughout the selected time points, specifically, 1, 3, 5, and 7 days, seeded collagen-coated HB scaffolds demonstrate a homogenous distribution of viable cells (Figure 4). Interestingly, the geometry of the structure substantially enhanced cellular migration on all the individual layers. Moreover, the grid-like architecture allowed for nutrient-rich α-MEM media to adequately reach cells located at the core of the scaffold. This section may be divided by subheadings. It should provide a concise and precise description of the experimental results and their interpretation, as well as the experimental conclusions that can be drawn.

### 3.2. Proliferation

**Figure 5:**
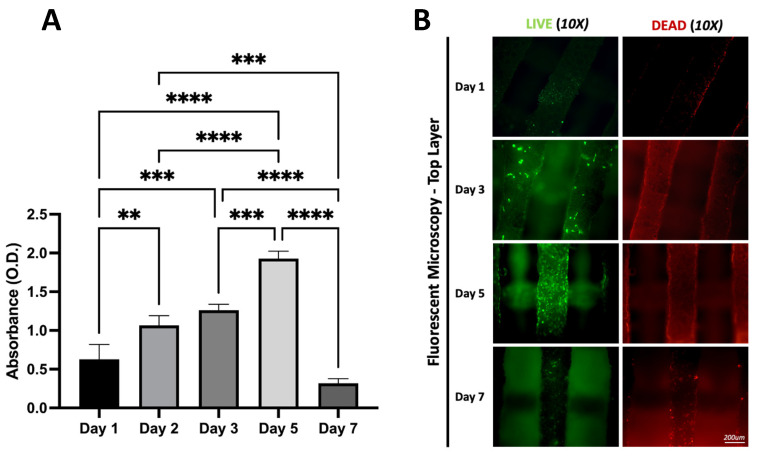
(**A**) MTS Assay of cells across 7 days confirms successful cell proliferation up to day 5, with an absorbance of 1.92 nm, followed by a rapid decrease of 0.31 nm at day 7. ** *p* ≤ 0.01, and *** *p* ≤ 0.001, **** *p* ≤ 0.0001. (**B**) LIVE/DEAD staining of cell-seeded scaffolds after 1, 3, and 5 days shows the greatest number of live cells on day 7 while the highest numbers of dead cells can be observed on day 7.

LIVE/DEAD staining of the HB scaffolds on day 1, demonstrated the presence of viable cells on the HB scaffolds. By day 3, the density of live cells increased, while migration ensured spatial distribution throughout the HB scaffold by day 5. Importantly, there were only a few fluorescently marked dead cells. The results were verified quantitatively using the MTS assay and are shown in Figure 5. By day 7, the number of cells decreased dramatically as a result of scaffold degradation.

**Figure 6:**
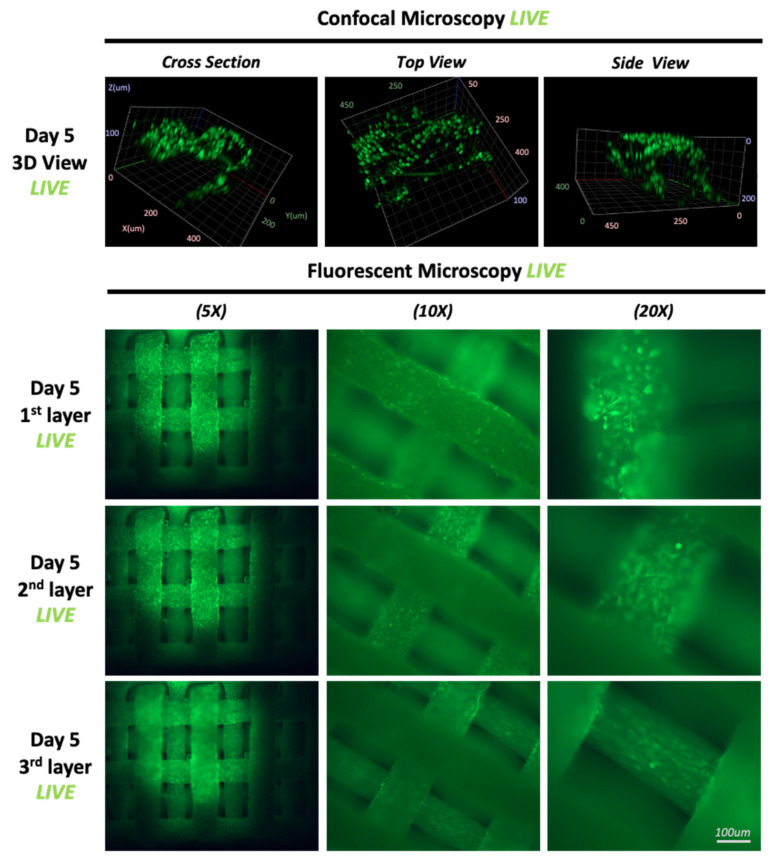
LIVE staining of cell-laden scaffolds at day 5. Cells form monolayers on the individual scaffold fibers. Quantification of LIVE fluorescence over 5 days indicates cell growth.

Specifically, cell proliferation peaked on day 5 (Figure 5), as cells developed monolayers on the individual fibers of the HB scaffold (Figure 6). Using different focal plains, fluorescent images of the top three layers show viable MG-63 cells homogeneously adhered to the scaffold. Clusters of cells were evident as cell proliferation continued, confirming the scaffold’s biocompatibility. However, confocal imagining shows that cellular proliferation occurs mostly on the external scaffold fibers. No cells were observed within the core of the scaffold’s individual fibers. Notably, up to day 5 in the culture, the HB scaffold provided adequate structural support, successfully maintaining its mechanical characteristics.

However, scaffold degradation begins to occur around day 6. The HA and poly fibers became brittle and developed microscopic fissures. This phenomenon was initially located close to the edges of the construct. However, from day 7 onwards, the HB scaffold degraded rapidly, and even minimal mechanical stress (movement) produced significant structural defects. Due to severe fragmentation, the number of surviving cells drastically decreased, as indicated by the MTS assay (Figure 5).

### 3.3. Cell Migration

**Figure 7:**
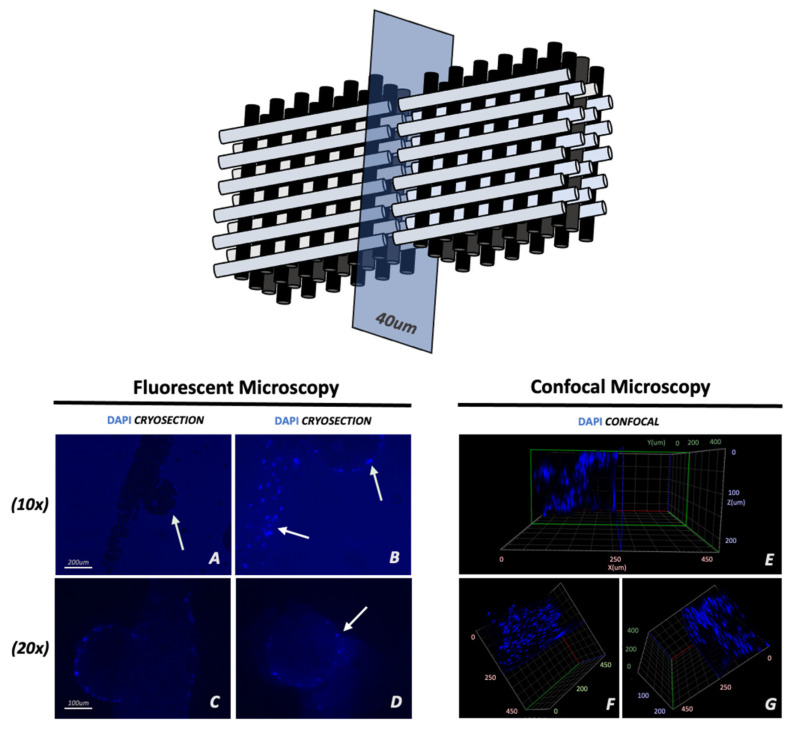
DAPI-stained cryotome sections of cell-seeded scaffolds on day 5 of culture confirm successful cell proliferation on the outer lamina of the scaffold. Top schematic indicates the slices obtained thought the scaffold. Arrows mark confluent cells on the fiber surface (**B**) and around the fibers (**C**,**D**). No cells were observed penetrating the core of the fibers (**A**) as an indirect indicator of low porosity. Confocal imaging demonstrates the formation of a cellular layer on the surface of the structure (**E**–**G**).

Sections of cell-seeded scaffolds were stained with DAPI at the end of 5 days in culture since that was the highest rate of proliferation observed (Figure 7). A thin layer of cells was present on the scaffold’s outer lamina attached to the collagen-coated surface. However, no cells penetrated the core of the fibers. To further support these findings, confocal microscopy was used to evaluate cellular morphology and distribution (Figure 7). A coronal three-dimensional view of the HB fibers depicted the same spatial distribution noted by cryosection analysis. Remarkably, results were consistent throughout the different levels of the structure, as cell growth can be identified up to the core levels of the complex geometrical construct.

### 3.4. Gene Expression

**Figure 8:**
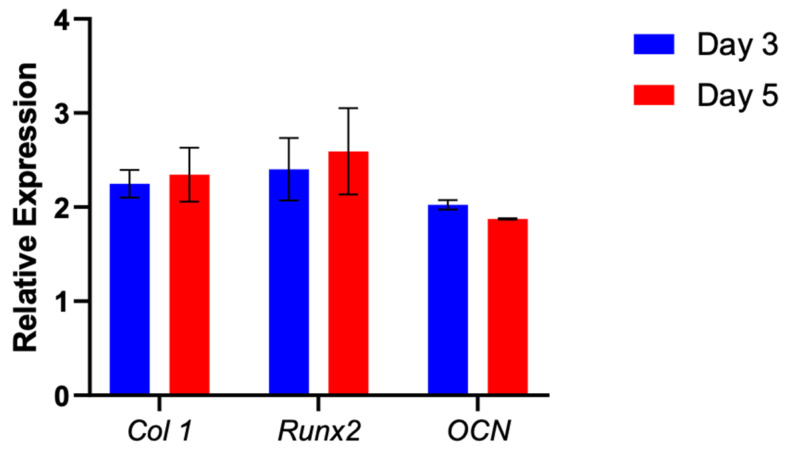
Expression of osteogenic markers Col I, OCN, and RunX-2 was measured by qRT-PCR and compared between day 3 and day 5 of incubation. Relative expressions were normalized to 18S (housekeeping gene). Gene expression showed a gradual increase throughout the 2 measured time points. Data are presented as mean ± SD (*n* = 5/timepoint).

mRNA levels of bone differentiation markers expressed by MG-63 cells were shown to be upregulated throughout the experiment. Specifically, we examined the temporal expression of Col I, OCN, and RunX-2 after 3 and 5 days of incubation. (Figure 8) All three genes showed stable expression at both 3 and 5 days post-plating, indicating that the cells have retained their osteogenic potential.

## 4. Discussion

Restoring bone structure and function in damaged bone tissue represents a monumental challenge for physicians and researchers. The biological complexity of bone fracture/defect healing is morphologically characterized by a cascade of well-orchestrated events, such as the inflammatory response, chondrogenesis, and osteogenesis [38]. However, internal repair mechanisms often lack the capacity to heal large structural defects, making bone grafting one of the few available therapeutic approaches. Autogenous cancellous bone grafts provide osteogenic and osteoconductive properties but the high incidence of morbidity during graft harvest and the high socioeconomic costs associated with this intervention create an urgent demand for alternative therapeutic approaches. Techniques such as 3D and 4D bioprinting represent a rapidly evolving niche that provides innovative and cost-efficient solutions for regenerative medicine [39]. Several studies have investigated shape-fitting polymers for both in vitro [20,21,40,41] and in vivo applications [22,23,40,42]. While the complex geometric structure of 3D scaffolds provides mechanical support, the use of an osteoinductive material, such as hydroxyapatite, tricalcium phosphate calcium sulfate, or bioactive glass-ceramics, is mandatory for the recruitment of immature cells and osteogenesis [15]. Therefore, selecting the appropriate structural design, biomaterial, and cellular or molecular components is crucial for successful bone regeneration.

In this study, we evaluated the spectrum of advantages provided by 3D-biomimetic implants. The constructs, developed by “Dimension Inx” are novel, as they are commercially available and “ready to print”, hence requiring little preparation prior to implanting. Recent advancements in bio-inks mean that the geometry and architecture of 3D-biomimetic structures can now provide increased resistance to tensile force while maintaining biodegradability. The balance between mechanical strength, porosity, and vascularization is paramount for the success of bone constructs. What sets our study apart is the unique focus on architecturally intricate HB scaffolds which, compared to other constructs, have an eight-layer grid-like design that offers high flexibility while maintaining a certain degree of stability. The use of such a complex geometrical shape allows “room” for cellular migration and vascular development while notably, requiring no need for crosslinking. Another unique element of this study centers on the size of the samples. This approach distinguishes the study by offering valuable insights into the possible constraints of complex 3D-printed HB scaffolds when addressing smaller-sized defects, primarily attributable to structural degradation and poor long-term adhesion. Previous in vitro experiments using similar HB have demonstrated that it serves as an ideal platform for studying cell behavior and cellular response in a controlled environment [43]. For example, HB has been shown to exhibit remarkable osteogenic potential as it produced a significant osteogenic response in adult human mesenchymal stem cells, without the need for any additional osteoinductive factors [21]. The 3D-biomimetic structure can serve as a physiologically relevant microenvironment for cells. However, one limitation of biomimetic materials is they are sometimes represented by low cellular adhesion. Namely, alginate or chitosan structures have low bioactivity due to the lack of cell-binding sites [15]. This issue was encountered while using HB, as the structure alone did not promote significant cellular adhesion in preliminary trials. In a previous study, Vandrovcová et al. in 2011 proved that coating synthetic scaffolds with collagen improves adhesion, proliferation, and osteogenic differentiation in MG-63 cells [44]. Moreover, studies show that the use of ECM proteins offers a “softer” substrate for cell adhesion while increasing osteogenic gene expression [45]. To circumvent this limitation, we tested whether a collagen-type I coating applied to the HB structure can improve cell adhesion. While a prominent formation of mineralized nodules was noted in similar scaffolds [46], no such clusters were identified in our study, probably due to the limited culture time. During the initial 5 days of the experiment, uniform and gradual cellular growth was evident, characterized by the formation of a cellular monolayer as observed through light and confocal microscopy. Expression analysis showed an upregulation in Col Type I, OCN, and RUNX two genes, indicating maintenance of osteogenic potential. Through its enhanced biomimicry of native bone, HB can facilitate proliferation, differentiation, and osteogenic potential for a limited interval. 

While ceramic-based bone constructs have been used in a large number of studies [46,47], research is still needed to develop scaffolds based on bioresorbable polymers [16]. Previously, we showed that SCPP polymerized alginate/collagen hydrogels facilitate MC-3T3 osteoblast growth and mineralization [48]. However, additional factors such as rigidity and essential blood supply are fundamental, especially for bone defects larger than 5 cm [49,50]. As such, adequate material porosity represents a vital characteristic of 3D-printed bone-mimicking scaffolds [51,52,53]. Interestingly, similar 3D-printed HB scaffolds have been used to serve as comprehensive platforms for developing synthetic-based in vitro vascularized bone grafts [20]. A recent study concluded that sequential seeding of hSMCs and HUVECs over a period of 3 weeks can generate early microvascular networks which can further develop into formed lumen structures throughout the 3D-printed HB. However, in our study, although initially maintaining adequate rigidity, the hydroxyapatite and poly bio-ink undergo degradation after only 7 days in culture. As we observed its degradation kinetics, we noted that structural disintegration might be attributed to the initial sectioning of the provided sheet. Furthermore, the relatively small size of the fragments used could be considered a contributing factor to rapid structural disintegration. Unlike other ceramic-based materials, the quick loss of strength during in vitro manipulation and the poor degradation of its internal structure makes HB scaffolds problematic for cellular development. Long-term survival and proliferation of cells were affected by this rapid fragmentation. Notably, between days 5 and 7, the structure became brittle and began to develop micro-fissures. By day 7, the HB scaffold was degrading rapidly, as even minimal mechanical stress would produce significant structural defects, leading to decreased cell counts. 

Further, a major limitation of our study was represented by poor cellular migration to the core of the HB fibers. Although the grid-like structure supported cellular proliferation throughout the eight individual layers, inks, such as alginate, matrigel, or hyaluronic acid, form a gelatinous protein mixture that can be used for the encapsulation of various cell types. The use of dense hydroxyapatite and poly ink severely limits cellular migration. DAPI-stained cryosections and confocal imaging showed that cells were unable to penetrate the 3D fibers. Even at peak cell proliferation, no cells were identified in the internal core of the individual fibers. However, promising findings were evident in other in vivo studies. For example, in murine experiments, subcutaneous HB implants demonstrated biocompatibility, improved tissue growth, and structure over commonly implemented hot-melt polymer-calcium phosphate composite materials [54]. Similar beneficial observations were reported in a rat posterolateral spinal fusion model, where HB was equally efficacious at promoting bone growth, as allograft-derived demineralized the bone matrix [42]. A case study of HB implantation in a calvaria bone defect in a rhesus macaque demonstrated that the synthetic graph can be quickly produced on a relevant scale and fashioned intraoperatively to press-fit into the defect site [22]. Finally, in a separate investigation, the viability of human adipose-derived stem cells (ADSCs) transduced with a lentiviral (LV) vector to overexpress BMP-2 was evaluated when loaded onto an HB scaffold. The study demonstrated successful BMP-2 production by transduced ADSCs on the HB scaffold, leading to significant bone formation in a hind limb muscle pouch model [24]. Histological analysis confirmed the formation of woven bone, which was notably absent in the control groups. Despite promising results observed in previously described settings, the limitations observed in our current experiment require additional investigation. Although novel bio-ink formulation, in conjunction with 3D and 4D-bioprinting technology, has great potential, choosing the correct formula for bio-inks is heavily dependent on the type of bone defect [39,55]. Future investigations are necessary as the loss of mechanical strength and reduced permeability might essentially lead to poor contact between the scaffold and defect margins. Arguably, the size of the implants could be fundamental for structural degradation, cell recruitment, angiogenesis, and regenerative healing, as 3D-HB implants might be better suited for in vivo applications on large bone defects. 

In conclusion, our study underscores the importance of gaining a deeper understanding of the dynamic components and scaffold functions for the advancement of future bio-inks in regenerative medicine. While our investigation yielded promising results, several limitations necessitate attention for further progress. Notably, the quest for long-term stability and mechanical integrity remains fundamental and must be addressed to ensure the practical viability of these scaffolds in clinical settings. Existing biomedical scaffolds could provide a temporary template for tissue engineering while the encapsulation of growth factors, cells, or even vascular components should be considered. The creation of precise mechanical and biological constructs for individual applications in bone tissue regeneration can, however, present significant challenges due to the complex and unique characteristics of bone and its diverse responses to different types of trauma and healing processes. 

Another limitation of the current study includes the absence of a control group utilizing different bio-ink formulations. This underscores the need for future research to provide a more comprehensive assessment of various biomaterials. Moreover, the translational issue remains significant, as no fully functional bone structure has yet been fabricated using 3D or 4D printing approaches.

## 5. Conclusions

The proposed study examined the biocompatibility, biodegradability, and osteoinductive capacity of a 3D-printed HB scaffold seeded with MG-63 cells in vitro. Initial adhesion and proliferation were successful but were unfortunately limited by the gradual degradation of the HB scaffold. The addition of Collagen Type 1 coating enhanced the robust adhesion of osteogenic cells, which successfully formed monolayers throughout the eight individual levels of the scaffold. However, cells did not penetrate the 3D-printed fibers, limiting their growth and migration to the external lamina. Notably, the cells exhibited osteogenic activity, as shown by increased Col I, RUNX 2, and OCN expression. Additionally, the geometric assembly of HB offers structural support and easy nutrient-rich media flow between individual layers. A certain degree of elasticity might make the HB structure adequate to fit complex defects. However, by day 7, microfractures and general degradation of the biomimetic material resulted in a substantial decrease in live cells. If controlled, HB cell-seeded grafts can be valuable in treating bone microlesions, providing temporary mechanical support and osteoinductive stimulus for dynamic in vivo healing. Overall, the study demonstrates the effectiveness of utilizing ECM-based proteins to facilitate the adhesion, growth, and migration of cells on synthetic polymer scaffolds and broadly outlines the osteoinductive characteristics of cell-seeded biodegradable constructs. 

## Figures and Tables

**Table 1:** Primers used for qPCR: Col I collagen type I, OCN osteocalcin, RunX-2 runt-related transcription factor 2.

Gene	Accession #	Forward	Reverse	Annealing Temp	Amplicon Size (bp)
Col 1	XM_054315083	5′-CCGCCGCTTCACCTACAGC-3′	5′-TTTTGTATTCAATCACTGTCTT-3′	64 °C	83
OCN	NM_199173	5′-AGCAAAGGTGCAGCCTTTGT-3′	5′-GCGCCTGGGTCTCTTCACT-3′	64 °C	63
RUNX-2	NM_001278478	5′-ATTCCTGTAGATCCGAGCACC-3′	5′-GCTCACGTCGCTCATTTTGC-3′	64 °C	81

## Data Availability

The data presented in this study are available on request from the corresponding authors.
